# Course and Outcome of Erythema Migrans in Pregnant Women

**DOI:** 10.3390/jcm9082364

**Published:** 2020-07-24

**Authors:** Vera Maraspin, Lara Lusa, Tanja Blejec, Eva Ružić-Sabljić, Maja Pohar Perme, Franc Strle

**Affiliations:** 1 Department of Infectious Diseases, University Medical Center Ljubljana, Japljeva 2, 1525 Ljubljana, Slovenia; vera.maraspin@kclj.si; 2 Department of Mathematics, Faculty of Mathematics, Natural Sciences and Information Technologies, University of Primorska, Glagoljaška 8, 6000 Koper, Slovenia; lara.lusa@famnit.upr.si; 3 Institute for Biostatistics and Medical Informatics, Medical Faculty, University of Ljubljana, Vrazov trg 2, 1000 Ljubljana, Slovenia; maja.pohar@mf.uni-lj.si; 4 Department of Perinatology, University Medical Center Ljubljana, Šlajmerjeva ulica 6a, 1525 Ljubljana, Slovenia; tanja.blejec@gmail.com; 5 Institute of Microbiology and Immunology, Faculty of Medicine, University of Ljubljana, Zaloška 4, 1000 Ljubljana, Slovenia; eva.ruzic-sabljic@mf.uni-lj.si

**Keywords:** erythema migrans, Lyme borreliosis, gestation, pregnancy outcome, *Borrelia burgdorferi* sensu lato

## Abstract

Information on Lyme borreliosis (LB) during pregnancy is limited. In the present study, the course and outcome of erythema migrans (EM) in 304 pregnant women, diagnosed in the period 1990–2015, was assessed and compared with that in age-matched non-pregnant women. The frequency of unfavorable outcome of pregnancies was also evaluated. The pregnant women reported constitutional symptoms less frequently than the non-pregnant women (22.4% vs. 37.2%, *p* < 0.001). Pregnant women diagnosed with EM later during pregnancy had a lower probability of reporting constitutional symptoms (odds ratio = 0.97 for 1-week difference in gestation week at diagnosis of EM, 95% CI: 0.94–0.99, *p* = 0.02). The outcome of pregnancy was unfavorable in 42/304 (13.8%) patients: preterm birth in 22/42 (52.4%), fetal/perinatal death in 10/42 (23.8%), and/or anomalies in 15/42 (35.7%). Several patients had potential explanation(s) for the unfavorable outcome. In conclusion, the course of early LB during pregnancy is milder than in age-matched non-pregnant women. The outcome of pregnancy with the treatment approach used in the present study (i.v. ceftriaxone 2 g once daily for 14 days) is favorable.

## 1. Introduction

Lyme borreliosis (LB) usually presents as the skin lesion erythema migrans (EM). The lesion, which is the result of tick bite inoculation of *Borrelia burgdorferi* sensu lato (s.l.) into the skin, develops early in the course of the disease. The causative agent can disseminate in some patients, resulting in secondary skin lesions and involvement of the nervous system, joints, heart, and/or eye [1].

Information on LB during pregnancy is limited. According to general belief, there are no differences in the course of the disease in pregnant and non-pregnant women. However, a PubMed literature search has found no straightforward data on the course of the infection in pregnant women, and information on the outcome of their pregnancies is limited [2,3,4,5,6,7,8,9,10,11,12,13,14,15,16,17,18,19,20,21,22,23].

The aim of our study was to evaluate and compare the course and outcome of early LB in non-pregnant and pregnant women and to assess the outcomes of the pregnancies.

## 2. Patients and Methods

### 2.1. Selection of Patients

Prospectively acquired data on women with EM who were examined at the LB Outpatient Clinic of the Department of Infectious Diseases, University Medical Center Ljubljana, Slovenia, in the period 1990–2015 were analyzed. All pregnant women diagnosed with EM according to the European criteria [24] were enrolled in the study.

### 2.2. Clinical Evaluation

Patients were evaluated before commencing treatment with antibiotics, again two weeks later, and then at two, six, 12, and 18 months. Pre-treatment characteristics were assessed using a standardized questionnaire that included demographic, epidemiologic, and clinical data as follows: the number, location, appearance, and diameter of EM; the presence of symptoms at the site of EM (itching, burning, pain); constitutional symptoms (fatigue, headache, myalgia, arthralgia, dizziness, nausea, fever) defined as complaints that newly developed or intensified since the beginning of EM, and the presence of extracutaneous manifestations of LB.

### 2.3. Laboratory and Microbiological Evaluation

Basic laboratory tests were performed at the first visit and two weeks later.

Serum antibodies to *B. burgdorferi* s.l. were determined at baseline and at follow-up examinations using either an indirect immunofluorescent assay with a local isolate of *B. afzelii* as antigen [25] or an indirect chemiluminescence immunoassay (LIAISON^®^) with antigens OspC and VlsE for the detection of IgM antibodies and VlsE for IgG antibodies.

As of 1994, a sample of citrated blood (5 mL until 2000, 9 mL subsequently) was taken at presentation for cultivation of borreliae in modified Kelly Pettenkofer medium [26]. Isolates were identified to species level using pulsed-field gel electrophoresis after *MluI* restriction of genomic DNA or by PCR-based restriction fragment length polymorphism of the intergenic region [27,28].

### 2.4. Treatment

At the first visit, patients received either i.v. ceftriaxone 2 g once daily, i.v. penicillin G 10,000,000 units twice daily, or oral phenoxymethylpenicillin 1 g three times per day. The duration of antibiotic treatment was 14 days.

### 2.5. Assessment of Outcome

The course and outcome of early LB in the pregnant women and the frequency of unfavorable outcome of their pregnancies (appraised by fetal death, pre-term birth, offspring malformations) were evaluated. The medical history was taken at each visit, including information on the presence of subjective symptoms, and patients were examined clinically. A gynecologist regularly monitored the course of gestation. At the first visit after delivery, detailed information about the birth and the infant was collected. A pediatrician monitored the babies at birth and after 6 months; however, several babies had more frequent and/or longer follow-ups.

### 2.6. Control Group

The course and outcome of EM in pregnant women was compared with that in age-matched non-pregnant woman diagnosed with EM at our institution in the same year.

### 2.7. Statistical Analysis

The characteristics of the pregnant women and the control group were compared using the Mann–Whitney test for numeric covariates and Fisher’s exact test for categorical covariates. Categorical variables were summarized with frequencies and percentages and 95% confidence intervals (CI), numeric variables with medians and interquartile ranges. To control for false positives, the *p* values shown in Table 1 were adjusted using a multivariate permutation procedure [29].

The association between gestation week at diagnosis of EM and the presence of constitutional symptoms was investigated using a logistic regression model. A possible departure from the assumption of a linear association between gestation week at which EM was diagnosed and the logit of probability of reporting the symptoms was assessed by fitting an additional model that included a restricted cubic spline transformation (with five knots) of the covariate.

The risk of an adverse outcome of pregnancy varies with week of gestation, and this was used as the timescale. We compared the risk for women in the same gestation week with respect to gestation week at diagnosis of EM. Four additional preselected possible confounders (age, duration of EM at diagnosis in weeks, multiple EM or borreliae isolated from blood, presence of constitutional symptoms) were included in univariable and multivariable Cox regression models.

To account for a possible selection bias of the pregnant patients, 39 patients enrolled in the first eight weeks of pregnancy were included in the risk set only after week 9 of their pregnancy.

R statistical language was used for all the analyses [30].

### 2.8. Ethical Considerations

The study was performed according to the Declaration of Helsinki and was approved by the Medical Ethics Committee of the Ministry of Health of the Republic of Slovenia (35/04/09). The Ethics Committee waived the need for written informed consent.

## 3. Results

Among 14,010 patients diagnosed with EM at our institution during a 26-year period, 307 were pregnant, and, for 304 (99%), the natural outcome of the pregnancy was available; 105 of these 304 patients have been reported previously [18,20,23].

### 3.1. Pre-Treatment Characteristics

**Figure 1:**
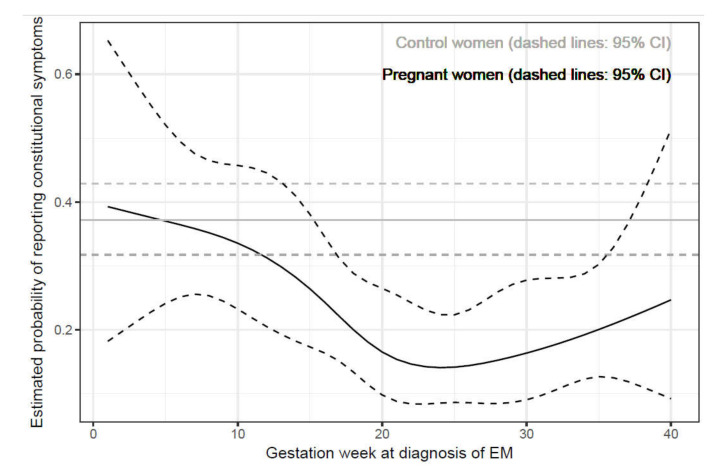
Estimated probability of reporting constitutional symptoms in pregnant and non-pregnant women with erythema migrans. EM, erythema migrans.

In the comparison of basic clinical characteristics of pregnant and non-pregnant women with EM, findings were analogous for the majority of parameters, with the exceptions that the pregnant women less often had a ring-like EM (42.4% vs. 55.3%, *p* = 0.002), less often had EM located on the trunk (14.1% vs. 24.0%, *p* = 0.009), and less often reported constitutional symptoms (22.4% vs. 37.2%, *p* < 0.001). When the analyses were adjusted for multiple comparisons, the differences remained significant for ring-like EM and the presence of constitutional symptoms (Table 1). Patients who were diagnosed in the later stages of pregnancy had a lower probability of reporting constitutional symptoms (OR = 0.97 for 1-week difference in gestation week at diagnosis of EM, 95% CI: 0.94–0.99, *p* = 0.02). Our data do not prove a non-linear association between gestation week and the logit of probability (*p* = 0.20). When including the non-linear term, the estimated prevalence of constitutional symptoms decreased very markedly only in the first 20 weeks of pregnancy but afterwards remained low (Figure 1).

In both groups of women, the proportion of patients with borrelial IgM and/or IgG serum antibodies at presentation (63/295, 21.4% vs. 72/289, 24.9%), the isolation rate of borreliae from blood (8/216, 3.7% vs. 4/187, 2.1%), and the ratio between the isolated *Borrelia* species (six *B. afzelii* and two *B. garinii* vs. three *B. afzelii* and one *B. garinii*) were similar.

At the initial visit, dissemination of borreliae was identified in 22 pregnant women: 14/304 (4.6%) had multiple EM; *B. burgdorferi* s.l. was isolated from the blood in 8/216 (3.7%) patients (all had solitary EM).

### 3.2. Treatment

Of 304 patients, 299 (98.4%) were treated with i.v. ceftriaxone 2 g once daily, three (1.0%) were treated with i.v. penicillin G 10,000,000 units twice daily, and two (0.7%) with oral phenoxymethylpenicillin 1 g three times per day.

### 3.3. Post-Treatment Course and Outcome

#### 3.3.1. Comparison of Pregnant and Non-Pregnant Women

After antibiotic treatment, the course and outcome of borrelial infection was uneventful in both groups of women: the EM disappeared in 14 (5–18) days in pregnant women and in 11 (3.5–15) days in non-pregnant women (*p* = 0.25). None of the 304 patients in either group developed any new objective manifestation of LB following treatment. Persistence of EM, defined as visible EM at follow-up 2–3 months after the beginning of antibiotic treatment, was found in 3/304 (1%) patients in each group. At the 2-month visit, only 5/304 (1.6%) pregnant women reported symptoms that newly developed or intensified after the beginning of EM; at 6 months, 1/300 (0.3%) reported such symptoms. The corresponding findings for the control group were 2/293 (0.7%) and 0/287, respectively.

#### 3.3.2. Outcome of Pregnancies

The outcome of pregnancy was unfavorable in 42/304 (13.8%) patients: preterm birth in 22/42 (52.4%), fetal/perinatal death in 10/42 (23.8%), and/or anomalies detected at birth or within the first year of life in 15/42 (35.7%). Several patients had a potential explanation for the unfavorable outcome (Table 2). At a significance level of alpha = 0.01, none of the chosen factors were associated with a higher risk of unfavorable outcome (Table 3).

## 4. Discussion

Several mechanical and pathophysiologic changes occur during pregnancy, and immune adaptations develop to accommodate the fetus [31]. As pregnancy progresses, hormone levels (estradiol, progesterone) increase markedly in association with several immunologic changes, including a shift from Th1 to Th2 immunity, which results in decreasing robustness of cell mediated immunity [32]. Consequently, the acquisition, clinical presentation, and course of infectious diseases in pregnant women may be altered [33]. Although clinical findings indicate that the course of most infections in pregnant and non-pregnant women are similar, some diseases (for example, listeriosis and malaria) are more frequent during gestation, and several (such as influenza, hepatitis E, herpes simplex virus infections, malaria) are more severe in pregnant women [31]. While it is well known that some non-infectious diseases, such as multiple sclerosis, have a milder course during pregnancy [34], no infectious disease has been reported to have a less severe course during gestation. There is a concept that pregnancy does not affect the course and outcome of early LB; however, studies that directly compare the course of LB in pregnant and in non-pregnant women are lacking.

Our study has shown that the majority of basic clinical and epidemiologic characteristics of EM before treatment with antibiotics were analogous in the two groups of women and consonant with previous findings in Slovenian patients with EM [35,36,37,38,39,40,41,42] and that the outcome after antibiotic treatment was excellent regardless of pregnancy. No subsequent objective manifestations of LB were established in either of the two groups, and the proportion of patients with symptoms at follow-up visits was even lower than found in other recent studies from Slovenia [37,38,39,40,41,42], possibly because only young, previously healthy patients were included in the present study.

Nevertheless, there were also several differences. We do not have a reliable explanation for the observation that the pregnant women less often had ring-like EM despite similar duration of the skin lesion before treatment, but we stress that the findings in our control group are in agreement with previous reports in Slovenian patients with EM [35,36,37,38,39,40,41,42]. Furthermore, the proportion of reported constitutional symptoms accompanying EM was lower in the pregnant women, indicating that the course of EM during pregnancy was milder than in the age-matched non-pregnant women (Table 1, Figure 1), as also shown in previous reports on EM from the same region [35,36,37,38,39,40,41,42]. Our results indicate that the probability of reporting constitutional symptoms systematically decreases with gestation week at diagnosis of EM (EM was diagnosed a median 7 days after the appearance of the skin lesion) and that women infected during the later stages of pregnancy report fewer constitutional symptoms compared with those infected during the early phases of pregnancy, who are more similar to non-pregnant women.

Since *B. burgdorferi* s.l. does not produce its own toxins or extracellular matrix-degrading proteases, most manifestations of LB result from inflammation generated by the host immune response to the spirochete [43]. Thus, fewer symptoms, as found in the present study of pregnant women with EM, may be associated with lower levels of inflammation. This assumption parallels findings in animal models where, for example, Lyme arthritis in pregnant mice was less severe. The amelioration of arthritis was associated with a shift in inflammatory responses—that is, the down-regulation of Th1 responses, most likely via the progesterone-mediated up-regulation of Th2 cytokine production, resulting in the reduction in pathogenic inflammatory responses during gestation in the mice [44]. In addition, our previous work has shown that higher numbers of constitutional symptoms in EM patients are associated with greater Th1 or Th17-associated cytokine responses, which is consistent with findings that immune responses shift towards a Th2 response in the course of pregnancy [45].

Information on the outcomes of pregnancy in women who develop LB during gestation is limited [46]. Findings of a PubMed literature search for the period of 1985 to January 2020 are shown in Table 4. The search found several individual case reports and a few series on unfavorable outcome of pregnancy in patients who were treated or not treated with antibiotics, the large majority of studies being from the two decades after recognition of LB [2,3,4,5,6,7,8,9,10,11,12,13,14,15,16,17,18,19,20,21,22,23]. The described cases show no uniform pattern of abnormalities. In the majority of cases, only a temporal association with maternal LB was described but no causal relationship was confirmed or searched for, and, in some articles, the proof of borrelial infection was imprecise by present standards. In addition, in patients in whom spirochetes were identified microscopically or by culture of placenta or autopsied infants, no signs of inflammation, granuloma formation, or necrosis in the affected tissues were detected. Moreover, Mather et al. reported the absence of the transplacental transmission of Lyme disease spirochetes from reservoir mice to their offspring [47], though the human placenta could be different to that of the mouse. Thus, the association between maternal borrelial infection and unfavorable outcome of pregnancy remains unclear, and the risk of adverse outcomes from maternal Lyme borreliosis has been interpreted to be negligible.

In the present study, 42/304 (13.8%) patients had an unfavorable outcome of pregnancy. However, several of these patients had a potential explanation for the unfavorable outcome (Table 2), and none of the tested parameters were associated with unfavorable pregnancy outcome (Table 3). Although our multivariable analyses showed an association between the week of pregnancy in which EM was diagnosed and unfavorable outcome, suggesting that patients infected earlier during pregnancy might have a higher risk of such an outcome, the diminishment of the odds of unfavorable outcome with the duration of pregnancy is an expected finding that is valid for the overall population of pregnant women. Thus, unfavorable outcome of pregnancy in women who had LB during pregnancy does not in any way signify that borrelia infection was the cause of the adverse result. Furthermore, the frequency of unfavorable outcome of pregnancy as found in the present study is comparable to the findings described in the literature for the general population, with the exception that the miscarriage rate in our study (11.6%) is somewhat lower than in the majority of previous reports (15–20%) [48,49,50,51] yet still in agreement with a Swedish study [51]. The findings of the present study are also in accord with the pregnancy outcomes reported for the general Slovenian population in the corresponding time frame [52], including miscarriage rate (11.6% in the present study versus 12% in the general population), preterm birth (7.2% versus 6.1%), and anomalies detected at birth (2.6% versus 1.7%). However, we would like to stress that we did not perform a cohort study, where all women enter the study at the beginning of their pregnancy, and therefore we did not attempt to analyze the approximate prevalence of unfavorable outcomes by trimester or to compare them with values observed in the overall population of pregnant women. In our study, pregnant women were enrolled at the gestation week when they were diagnosed with EM and left the study at delivery or at the gestation week of an unfavorable event. Consequently, women experiencing abortion or early delivery were less likely to be included in the sample of pregnant women diagnosed with EM compared with those who did not experience such unfavorable events.

All but five pregnant women were treated for their EM with i.v. ceftriaxone for 14 days. According to current knowledge, this is clearly overtreatment of EM in non-pregnant and possibly also in pregnant women. However, 30 years ago, the understanding of LB was somewhat rudimentary, and information on the course and outcome of pregnancy in patients with early LB was limited to individual case reports, several of them indicating unfavorable outcomes after treatment of EM with oral antibiotics [3,10]. At that time, we decided on a treatment protocol that would achieve high enough levels of antibiotics not only in skin but also in the placenta and fetus; the decision being based on the premise that damage to the fetus probably results from the direct dissemination of borreliae or indirectly through damage to the placenta. In the years since, a concept has been developed in which the outcome of pregnancy with this treatment approach is similar to that in the general population [20]. However, because cases of LB during gestation are not numerous, we did not alter our approach and have been waiting to see whether studies from other research groups would confirm that the same outcome would follow oral antibiotic treatment, as recommended for EM in the non-pregnant population. A report published in 2010 (the most recent available information) states that the proportion of unfavorable pregnancy outcomes in patients with LB was the highest in patients who received no antibiotic treatment for their LB, followed by those who received oral antibiotics, and it was the lowest in patients treated with parenteral antibiotics [22]. The report, although it has several methodologic drawbacks, has influenced us to not change our treatment approach. The consequence of this “wait and watch” tactic is that we know that the outcome of pregnancies is relatively favorable using our treatment protocol, but we do not know whether the same results could be obtained with oral treatment, as is usually recommended for EM. Furthermore, since we did not demonstrate the direct detection of borreliae in fetal tissue or umbilical blood, etc., which is a substantial limitation of the present study, we do not know whether a relatively favorable outcome of pregnancy is the result of our efficacious antibiotic treatment or a consequence of very rare or perhaps even non-existent borrelial involvement in the offspring.

## 5. Conclusions

The course of early LB during pregnancy is milder than in age-matched non-pregnant women. The smaller proportion of pregnant patients reporting constitutional symptoms at the time of EM diagnosis might be the result of immunologic changes during gestation.

The outcome of pregnancy with the treatment approach used in the present study (i.v. ceftriaxone 2 g once daily for 14 days) is favorable. Multivariable analyses showed that patients who develop EM in the early stages of pregnancy might have a higher risk of unfavorable outcome.

## Figures and Tables

**Table 1:** Basic demographic, clinical, and laboratory characteristics of 304 pregnant women before antibiotic treatment of erythema migrans in comparison with 304 sex- and age-matched patients diagnosed with erythema migrans in the same year.

	Pregnant Women	Control Group	*p*	Adjusted *p*
No. of patients	304	304		
Age (years)	29.5 (27–33)	29 (26–33)		
Tick bite ^a^	191 (62.8; 57.1–68.3)	190 (62.5; 56.8–68.0)	>0.99	>0.99
Interval from bite to EM onset (days) ^b^	14 (7–24)	13 (8–21)	0.92	>0.99
Interval from EM onset to diagnosis and treatment (days)	7 (4–18)	8 (4–22)	0.10	0.55
Location of EM ^c^:			0.009	0.062
Extremities	256 (84.2; 79.6–88.1)	227 (74.7; 69.4–79.5)		
Trunk	43 (14.1; 10.4–18.6)	73 (24.0; 19.3–29.2)		
Head, neck	5 (1.6; 0.5–3.8)	4 (1.3; 0.4–3.3)		
Size of EM ^c^ (cm)	10 (7–16)	10 (7–17)	0.54	>0.99
Ring-like appearance of EM ^c^:	129 (42.4; 36.8–48.2)	168 (55.3; 49.5–60.9)	0.002	0.016
Local symptoms	170 (55.9; 50.1–61.6)	182 (59.9; 54.1–65.4)	0.37	0.97
Itching ^d^	154 (50.7; 44.9–56.4)	163 (53.5; 47.8–59.3)		
Burning ^d^	26 (8.6; 5.7–12.3)	33 (10.9; 5.6–14.9)		
Pain ^d^	26 (8.6; 5.7–12.3)	39 (12.8; 9.3–17.1)		
Constitutional symptoms	68 (22.1; 17.8–27.5)	113 (37.2; 31.7–42.9)	<0.001	0.002
Fatigue ^d^	29 (9.5; 6.5–13.4)	62 (20.4; 16.0–25.4)		
Headache ^d^	33 (10.9; 7.6–14.9)	62 (20.4; 16.0–25.4)		
Myalgias ^d^	12 (4.0; 2.1–6.8)	24 (7.9; 5.1–11.5)		
Arthralgias ^d^	18 (5.9; 3.5–9.2)	32 (10.5; 7.3–14.5)		
Fever ^d^	5 (1.6; 0.5–3.8)	8 (2.6; 1.1–5.1)		
Dizziness ^d^	7 (2.3; 0.9–4.7)	11 (3.6; 1.8–6.4)		
Multiple EM	14 (4.6; 2.5–7.6) *	16 (5.3; 3.0-8.4) **	0.71	>0.99
Other manifestations of LB ^e^	2	0		

Data are medians (interquartile range) or frequencies (percentage; 95% confidence interval). *p* values were obtained with Mann–Whitney tests for numeric variables and chi-squared Fisher’s exact tests for categorical variables. Abbreviations: EM, erythema migrans; LB, Lyme borreliosis. ^a^ At site of later EM skin lesion; ^b^ data for 189/191 pregnant and 185/190 non-pregnant women who recalled a tick bite at the site of later EM; ^c^ findings for the primary lesion in patients with multiple EM; ^d^ number (%) of patients reporting an individual symptom; ^e^ one patient with associated borrelial lymphocytoma, one with transitory cardiac conduction disorder; * number of skin lesions: 2–13 (IQR 2–6); ** number of skin lesions: 2–12 (IQR 2–3).

**Table 2:** Unfavorable outcome of pregnancy in 42 patients after antibiotic treatment of erythema migrans.

Pregnancy Trimester	Case No. (Year of Diagnosis)	Tick Bite	Onset of EM	Diagnosis of EM	Preterm Birth	Fetal ^a^/Perinatal ^b^ Death	Anomalies (Evident at Birth or Later ^c^), Other Abnormalities	Potential Explanations for Unfavorable Outcome
		Week of Gestation						
First trimester	1 (1998)	no	2	3		11 ^a^		
	2 (1996)	2	3	4			syndactyly	anomaly present in family members
	3 (2002)	3	4	4		16 ^a^		uterus septus
	4 (2008)	no	1	4		10 ^a^		*
	5 (1997)	3	5	5	33 ^d^			pre-eclampsia at week 31
	6 (1997)	no	2	5		10 ^a^		
	7 (1999)	−2 ^e^	4	5			lacrymal canal stenosis^c^	
	8 (2008)	2	5	6		10 ^a^		multipara (6 children, 2 spontaneous abortions previously) *
	9 (1996)	6 ^e^	−2 ^e^	6	25	25 ^b^		chorioamnionitis, vasculitis of umbilical vessels
	10 (2010)	4	6	7		10 ^a^		
	11 (1993)	no	6	7		9 ^a^		uterus bicornus
	12 (2013)	no	4	8	32 ^d^			
	13 (1997)	9	9	10	25	25 ^b^		
	14 (1996)	9	9	10	36			thyroiditis during pregnancy
	15 (2010)	8	9	10	35 ^d^			partus imminens (hospitalization from gestational week 24)
	16 (2006)	8	10	12	30 ^d^			preterm premature rupture of membranes
	17 (2008)	8	10	12	34 ^d^			
	18 (2009)	no	10	12		12 ^a^		
	19 (1997)	no	11	16			ureteral stenosis, hydronephrosis, hydroureter dex ^c^ (apparent 10 months after birth)	
	20 (2010)	no	11	16			fetal growth retardation, CVI in perinatal period with consequent spasticity, left ovarian cyst	
	21 (2001)	11	12	13	36			
**Summary**		**13/21 (62%)**	**6 (−2–12)**	**7 (3–16)**	**9/69 (13%)**	**10/69 (14%)**	**4/69 (6%)**	**9/69 (13%)**
Second trimester	22 (2005)	no	13	14	36			
	23 (2000)	12	15	16			stenosis of pulmonary arteria, open foramen ovale	
	24 (2004)	15	15	16	36		hypospadia	
	25 (1994)	12	15	16	36 ^d^		ASD, VSD	cervical insufficiency
	26 (1993)	no	15	16	36			
	27 (2001)	no	18	19	-		extrasystoles (shortly after birth, duration 3 weeks)	
	28 (2006)	16	20	20			ASD, open foramen ovale, stenosis of pulmonary artery ^c^ (apparent 3 months after birth)	
	29 (2000)	17	20	20			VUR	
	30 (2001)	18	19	20			hearing deficit	
	31 (1993)	19	20	21	26 ^d^			uterus septus; cervical insufficiency
	32 (1994)	18	19	22	36			
	33 (2010)	21	23	23	36		month: ASD	
	34 (2010)	no	23	24	36			
	35 (1992)	22	23	24	36			
	36 (2001)	no	24	30	36			*
	37 (2003)	21	25	25	36			
	38 (2004)	15	26	28	35 ^f^			
	39 (2004)	26	28	28	36			**
**Summary**		**13/18 (72%)**	**19 (13–28)**	**20 (14–28)**	**13/150 (9%)**	−	**8/150 (5%)**	**2/150 (1%)**
Third trimester	40 (1995)	31	32	32			5th month: VUR ^c^	
	41 (2007)	33	34	34			ASD, VSD, open ductus arteriosus Botalli	
	42 (1992)	no	32	35			7th month: bilateral ureteral stenosis, hydronephrosis ^c^	
**Summary**		**2/3 (67%)**	**32 (32–34)**	**34 (32–35)**	**0/85**	−	**3/85 (4%)**	**0/85**

Abbreviations: EM, erythema migrans; CVI, cerebrovascular insult; ASD, atrial septum defect; VSD, ventricular septum defect; VUR, vesicoureteral reflux. ^a^ Fetal death due to missed abortion (six patients) or spontaneous abortion (two patients); ^b^ live at birth, death occurred within few minutes; ^c^ later diagnosis of anomalies (within the first year after birth); ^d^ respiratory difficulties at birth; ^e^ weeks prior to conception; ^f^ respiratory distress syndrome and severe icterus at birth; * patient had multiple EM; ** isolation of *Borrelia afzelii* from blood.

**Table 3:** Factors associated with unfavorable outcome of pregnancy.

Covariate	Univariable Analysis HR (95% CI); *p*	Multivariable Analysis HR (95% CI); *p*
Gestation week at diagnosis (1-week difference)	0.97 (0.93–1.00); 0.067	0.95 (0.92–0.99); 0.013
Age (10-year difference)	1.35 (0.76–2.40); 0.31	1.43 (0.78–2.62); 0.25
Duration of EM before diagnosis (10 days difference)	0.82 (0.65–1.04); 0.11	0.79 (0.61–1.02); 0.07
Multiple EM or borreliae isolated from blood	1.52 (0.54–4.28); 0.43	1.88 (0.65–5.41); 0.24
Presence of systemic symptoms	0.53 (0.22–1.26); 0.15	0.46 (0.19–1.11); 0.08

Abbreviations: HR, hazard ratio; CI, confidence interval.

**Table 4:** Reports on unfavorable outcomes of pregnancy in patients with Lyme borreliosis during gestation.

Author(s) and Year Type of Study	Pregnant Women			Outcome of Pregnancy			
	Tick Bite Gestational Week of LB Onset/Diagnosis	LB Signs/LB Symptoms/Antibiotic Treatment Other Clinical Data	LB Serology	Preterm Birth: Week (Weight)	Fetal or Perinatal Death: Week (Weight)	Anomalies, Other Abnormalities	Evidence of *Borrelia burgdorferi* Sensu Lato Infection of Fetus or Child
Schlesinger et al. 1985 Case report	No 6–7/8	MEM, stiff neck/headache, malaise, arthralgias/no No	Positive	35 (ND)	35 (ND) Respiratory distress, death 39 h after delivery	Aortic valvular stenosis, patent ductus arteriosus, coarctation of aorta, tubular hypoplasia of aorta and aortic arch, endocardial fibroelastosis No	A few spirochetes in spleen, renal tubule, bone marrow seen in paraffin block sections stained using modified Dieterle method. No evidence of inflammation, necrosis, or granuloma in any organ. Placenta not available. Later examination: immunohistochemical detection of spirochetes in cardiac tissue
MacDonald 1986, 1989 MacDonald et al. 1987 Retrospective analysis of perinatal autopsies 1978–1985; prospective study on perinatal deaths 1985–1988	No 1–2/LB not diagnosed	EM, arthritis/no/no No	Positive	No	At term (2500 g) Stillbirth	VSD Retardation of intrauterine growth	Culture of spirochetes from fetal liver tissue; IFA detection of spirochetes in fetal liver, heart, adrenal glands, subarachnoid space; silver stains: spirochetes in myocardium, brain, liver, placenta. No inflammation in fetal tissues, rare plasma cells in isolated placental villi.
	No ND/LB not diagnosed	No/no/no Toxemia in w 17	Negative	19 (514 g)	19 (514 g) Stillbirth	ASD No	Culture of spirochetes from fetal liver tissue; IFA detection of spirochetes in fetal liver and placenta.
	ND ND/LB not diagnosed	No/arthralgias/no Toxaemia in w 22	Negative	23 (490 g)	23 (490 g) Stillbirth	Coarctation of aorta No	Culture of spirochetes from fetal liver tissue; IFA detection of spirochetes in fetal liver and placenta. No tissue inflammation.
	ND ND/LB not diagnosed	No/no/no No	Negative	15 (85 g)	15 (85 g) Stillbirth	No No	Culture of spirochetes from fetal liver tissue; IFA detection of spirochetes in fetal liver, placentaNo tissue inflammation
	ND ND/LB not diagnosed	No/no/no Vaginal bleeding in 1st trimester	ND	No	39 (2250 g) Respiratory distress, death in 4 h	VSD, hydrocephalus, meningomyelocele, omphalocele, spina bifida, club foot No	Immunohistochemical detection of spirochetes in fetal tissue
	ND ND/LB not diagnosed	No/no/no No	ND	No	40 (1950 g) Respiratory distress, death in 30 min	VSD, absent left hemidiaphragm Retardation of intrauterine growth, cardiac dysfunction	Indirect IFA detection of spirochetal fragments in fetal tissue
	ND ND/LB not diagnosed	No/no/no Vaginal bleeding in 2nd trimester	Negative	17 (30 g)	17 (30 g)	Hydrocephalus No	Indirect IFA detection of spirochetes in fetal brain
	ND ND/LB not diagnosed	No/no/no Vaginal bleeding in 2nd trimester	Negative	16 (150 g)	16 (150 g)	No No	Spirochetes identified in fetal brain using immunohistochemical technique with monoclonal antibodies. No inflammation found in fetal viscera
	ND ND/LB not diagnosed	No/no/no No	ND	12 (294 g)	12 (294 g)	No No	Culture of fetal viscera in BSK medium yielded *B. burgdorferi* and other bacteria from fetal kidney; immunohistochemistry negative for spirochetes in fetal viscera
	ND ND/LB not diagnosed	No/arthralgias, myalgias, headache/no No	Negative	25 (ND)	25 (ND) Intrauterine fetal death	VSD No	Indirect IFA detection of spirochetes in fetal tissue
	ND ND/LB not diagnosed	No/no/no No	ND	No	40 (3746 g)	No Neonatal sepsis, respiratory distress in 1st hour of life	Rare spirochetes found in “normal” placental villi
	ND ND/LB not diagnosed	No/no/no Toxemia in w 37	ND	37 (2157 g)	No	No Neonatal sepsis, respiratory distress in 1st day of life	Many spirochetes identified in placenta using Warthin–Starry silver impregnation technique
Markowitz et al. 1986 Retrospective study on 19 pregnant women with LB	ND 6/8	EM, stiff neck, arthritis/headache/penicillin V 10 days No	Positive	20 (ND)	20 (ND) Intrauterine fetal death	No No	Culture and indirect IFA negative. No inflammation found in fetal tissues.
	ND 10/LB not diagnosed	Facial palsy, arthritis/headache/no No	ND	36 (2100 g)	No	No Hyperbilirubinemia	ND
	ND 20/21	EM, stiff neck/headache, arthralgia/ erythromycin 10 days (in w 21), penicillin V 10 days (in w 27) No	ND	No	No	Syndactyly No	ND
	ND 27/27	EM/no/penicillin V 10 days No	ND	No	No	Cortical blindness Developmental delay	ND
	ND 39/39	EM, meningitis/no/no No	ND	No	No	No Generalized rash, hyperbilirubinemia	ND
Ciesielski et al.1987 Prospective study on 17 pregnant patients with LB	ND ND/4	Not specified/not specified/data on prescribed antibiotic not available ND	Positive	13 (ND)	13 (ND) Spontaneous abortion	No No	No evidence of borrelial infection on stains or cultures of fetal tissues.
	ND ND/7	Not specified/not specified/data on prescribed antibiotic not available ND	Positive	No	No	Syndactyly No	ND
Weber et al. 1988 Case report	Yes 10/12	EM/no/penicillin 1g tid 7 days No	Seroconversion	No	At term (3400 g) Respiratory distress, death 23 h after delivery	No No	Silver stain and monoclonal antibody identification of spirochetes in brain and liver. No significant inflammation found in any organ.
Andrásová et al. 1988 Case report	ND ND/23	EM, facial palsy/arthralgias, low fever/yes (antibiotic not specified) Vaginal bleeding in 1st trimester	Positive	32 (1450 g)	No	No Respiratory distress, anemia	Placenta: no spirochetes and no inflammation.
Nadal et al. 1989 Serologic study on 1416 pregnant women and their offspring	Yes 1st trimester/LB not diagnosed	EM, arthritis/no/no No	Positive	No	No	VSD No	ND
Lavoie et al. 1990 Case report	No ND/LB not diagnosed	No/arthralgia, malaise/no No	Negative	No	At term (ND)Respiratory distress, myocardial dysfunction, death 8 days after delivery	No No	Isolation of *B. burgdorferi* from frontal cerebral cortex; silver staining: spirochetes in brain and heart.
Hercogová et al. 1993, 1994 Prospective studies on 15 and 19 pregnant patients with EM	No 6/10	EM/arthralgia, paresthesias/penicillin V 5 days (in w 10), penicillin V retreatment (in w 14) No	Positive	15 (ND)	15 (ND) Intrauterine fetal death	No No	Borrelia-like organism in ultrathin sections of the decidua detected using monoclonal antibody H9724 against flagellin.
	Yes 16/18	EM/fatigue/ampicillin 21 days No	Positive	18 (ND)	18 (ND)	Spina bifida, hydrocephalus No	ND
	Yes 4/8	EM/no/penicillin V 24 days No	Negative	No	No	Unclosed ductus arteriosus Botalli No	ND
	Yes 25/30	EM/no/penicillin V 14 days No	Negative	No	No	Cryptorchidism (established at 2 years) No	ND
	ND ND/ND	EM/ND/yes (antibiotic not specified) No	ND	No	No	No Hypotrophia	ND
	ND ND/ND	EM/ND/yes (antibiotic not specified) No	ND	No	No	No Hyperbilirubinemia	ND
	No 18/24	EM/no/penicillin V 14 days, benzanthine penicillin No	Negative	No	No	Enamel defect No	ND
	No 31/33	EM/low fever/penicillin V 14 days No	Positive	No	No	Enamel defect No	ND
	Yes 20/23	EM/no/penicillin V14 days No	Positive	No	No	No Developmental delay	ND
Williams et al. 1995 Serologic study on 5011 newborns and their mothers	ND	ND/ND/yes (antibiotic not specified) ND	Positive	No	No	Hypospadia No	ND
Maraspin et al. 1996, 1999 Prospective cohort studies on 58 and 105 pregnant patients with EM	Yes Before conception/6	EM/no/ceftriaxone 2 g i.v. 14 days No	Negative	25 (610 g)	25 (610 g)Death in few minutes	No No	Warthin–Starry silver impregnation of fetal tissues: no spirochetes. No inflammation. Normal placenta. Chorioamnionitis and vasculitis of umbilical vessels.
	Yes 9/10	EM/no/ceftriaxone 2 g i.v. 14 days No	Negative	25 (450 g)	25 (450g)Death in few minutes	No No	Warthin–Starry silver impregnation of fetal tissues: no spirochetes. No inflammation. Normal placenta.
	Yes 5/5	EM/no/ceftriaxone 2 g i.v. 14 days Preeclampsia in w 31	Negative	33 (1720 g)	No	No Respiratory distress, hyperbilirubinemia	Not tested
	Yes 20/21	EM/no/ceftriaxone 2 g i.v. 14 days No	Negative	26 (840 g)	No	No Respiratory distress, bilateral ventricular and periventricular bleeding	Not tested
	Yes 23/24	EM/no/ceftriaxone 2 g i.v. 14 days No	Negative	36	No	No No	Not tested
	Yes 15/16	EM/no/ceftriaxone 2 g i.v. 14 days No	Negative	36 (2940 g)	No	ASD, VSD Respiratory distress, pneumothorax	Not tested
	No 32/35	EM/no/ceftriaxone 2 g i.v. 14 days No	Negative	No	No	Bilateral ureteral stenosis No	Not tested
	Yes 32/32	EM/no/ceftriaxone 2 g i.v. 14 days No	Negative	No	No	Vesicoureteral reflux No	Not tested
	No 11/16	EM/no/ceftriaxone 2 g i.v. 14 days No	Negative	No	No	Ureteral stenosis, hydronephrosis, hydroureter dex No	Not tested
	Yes 3/4	EM/no/ceftriaxone 2 g i.v. 14 days No	Negative	No	No	Syndactyly No	Not tested
	No 6/7	EM/no/ceftriaxone 2 g i.v. 14 days No	Negative	9 (ND)	9 (ND) Missed abortion	No No	Not tested
	No 2/5	EM/no/ceftriaxone 2 g i.v. 14 days No	Negative	10 (ND)	10 (ND) Spontaneous abortion	No No	Not tested
Lakos et al. 2010 Clinical experiences obtained using miscellaneous approaches	^a^ ND 1–5/ND	EM/ND/ND/ 4/6 pts oral antibiotic (ND on antibiotic type); 2/6 untreated	ND	8 (ND)—5 pts, 13 (ND)—1 pt	8–13 (ND) Spontaneous abortion	No No	ND
	ND 5/ND	EM/ND/No ND	ND	22 (ND)	22 (ND) Stillbirth	No No	ND
	ND 17/ND	EM/ND/cefuroxime axetil ND	ND	35 (ND)	No	No No	ND
	ND 13/ND	EM/ND/ceftriaxone 2 g i.v. 15 days ND	ND	No	No	No Small for date (2200 g at 39 w)	ND
	^b^ ND 13–27/ND	EM/ND/penicillin G i.v. or ceftriaxone iv ND	ND	No	No	Cavernous hemangioma No	ND
	ND/13/ND	EM/ND/ceftriaxone 2 g i.v. 15 days ND	ND	No	No	NoHyperbilirubinemia *	ND
	ND/38/ND	EM/ND/erythromycin 150 mg qid 30 days ** ND	ND	No	No	Dysplasia coxae Hyperbilirubinemia *	ND
	ND 18/ND	EM/ND/ceftriaxone 2 g i.v. 15 days ND	ND	No	No	Dysplasia coxae No	ND
	ND Before conception/ND	EM/ND/ceftriaxone 2 g i.v. 15 days ND	ND	No	No	Pyloric stenosis, dysplasia coxae No	ND
	ND 38/ND	EM/ND/No ND	ND	No	No	No Papulovesicular eruption at birth	ND
	ND 4/ND	EM/ND/No ND	ND	No	No	No Cerebral bleeding, developmental delay	ND
	ND 27/ND	EM/ND/No ND	ND	No	No	Hypospadia, cavernous hemangioma No	ND
	ND 13/ND	EM/ND/ceftriaxone 2 g i.v. 15 days ND	ND	No	No	Skeletal anomaly No	ND
Maraspin et al. 2011 Study on 7 pregnant patients with EM and borreliae isolated from blood	Yes 28/28	EM/No/ceftriaxone 2 g i.v., 14 days No	Negative	36 (2500 g)	No	No No	Not tested

Abbreviations: LB, Lyme borreliosis; MEM, multiple erythema migrans; EM, erythema migrans; ND, no data; pt, patient; pts, patients; VSD, ventricular septum defect; w, week; ASD, atrial septum defect; IFA, immunofluorescence assay; tid, three times daily; quid, four times daily. ^a^ Summarized data for six patients with spontaneous abortion; ^b^ summarized data for three patients with cavernous hemangioma; * exchange transfusion required; ** persistent EM, therefore patient treated after delivery with ceftriaxone 2 g i.v. for 15 days.
